# Slow Progression of Cognitive Dysfunction of Alzheimer's Disease in Sexagenarian Women with Schizophrenia

**DOI:** 10.1155/2015/968598

**Published:** 2015-07-12

**Authors:** Kazuo Sakai, Haruhiko Oda, Akira Terashima, Kazunari Ishii, Kiyoshi Maeda

**Affiliations:** Department of Physical Therapy, Takarazuka University of Medical and Health Care, School of Health Science, 1 Hanayashiki-Midorigaoka, Takarazuka, Hyogo 666-0162, Japan

## Abstract

Although both schizophrenia (SCZ) and Alzheimer's disease (AD) are among the most common psychiatric diseases, the interaction of these two is not well-understood. We investigated three women with SCZ who developed AD in their 60s. The patients presented with cognitive dysfunction such as loss of recent memory, which was confirmed by both clinical observations and neuropsychological tests. Their magnetic resonance and functional imaging findings were consistent with AD. Their brain atrophy advanced significantly during a 6-year observation period. However, their global cognitive function did not deteriorate significantly during this period. Although the cognitive reserve model might account for this discrepancy, our results suggest some interactions between the neuropathology of SCZ and AD and warrant further research.

## 1. Introduction

Schizophrenia (SCZ) is one of the most common chronic psychiatric disorders with a global prevalence of 0.5% to 1.5%. Alzheimer's disease (AD) is also a common disease, with a prevalence of 11% in males and 14% in females at 85 years of age [1]. Therefore, it is natural for some patients who suffered SCZ in their younger age to develop AD when they become older.

Nevertheless, not many studies have investigated the interaction between AD and SCZ. Most investigations of the two diseases have focused on the differential diagnosis of AD with delusion from late-onset SCZ or delusional disorder [2]. In addition, as originally termed dementia praecox by Kraepelin, some of the patients with SCZ show cognitive deterioration without the neuropathology of neurodegenerative disease [3, 4], which hampers AD diagnosis in patients with a history of SCZ. Furthermore, it is unclear whether a history of SCZ increases the risk for AD. Prohovnik et al. reported higher Alzheimer-type neuropathology in the elderly with SCZ [5]. However, other researchers argued against such a notion [6].

We investigated three patients with SCZ who developed AD in their 60s. All of them showed slower cognitive decline compared with average patients with AD. In this report, we try to verify the validity AD diagnosis in these patients and then discuss the interaction of the two diseases.

## 2. Case Presentation

### 2.1. Case A

**Figure 1:**
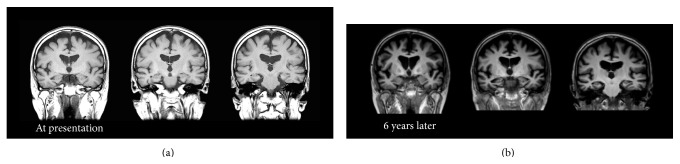
Coronal T1 weighted brain Magnetic Resonance Image (MRI) of the patient in Case A at presentation (a) and 6 years later (b). No remarkable vascular change or mass signs were observed. Diffuse cerebral and hippocampal atrophy, as well as enlargement of the lateral ventricles, which advanced at 6 years, was consistent with comorbid Alzheimer's disease.

**Figure 2:**
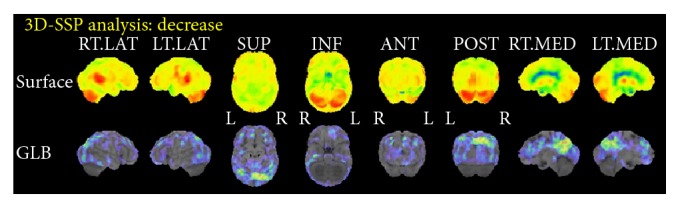
Three-dimensional stereotactic surface projections (3D-SSP) of single-photon emission computed tomography with N-isopropyl[^123^I]-p-iodoamphetamine (IMP-SPECT) of Case A. Marked decrease of blood flow at precuneus and posterior cingulate cortex were observed, which suggested the diagnosis of comorbid Alzheimer's disease.

A 66-year-old woman, who was diagnosed with SCZ in her twenties, presented to our clinic. After the first episode of SCZ, she got married and had two children. According to her family, she became emotionally unstable and made incoherent statements frequently and subsequently was brought for psychiatric examination. Her family reported that she was prescribed antipsychotics such as chlorpromazine or haloperidol. However, neither the duration nor doses of medication could be confirmed because of the lack of medical records. Although she did not function well socially, and her husband took her place in most of the social activities, her activity of daily living was still intact and she was able to do the housework until the age of 65. At 65, she was unable to use the shower, the facsimile, or other daily tools. She was also unable to recall recent conversations and began to ask the same questions repeatedly. Therefore, her family brought her to our dementia clinic. She had a history of hypertension and hyperlipidemia. At presentation, she had discontinued psychiatric treatment for more than a decade and was not prescribed any psychotropic medications. She was reluctant to receive any treatments and her speech was incoherent. She could not recall recent social or personal events and did not seem to remember the previous visit to the clinic. She could independently perform activities of daily living, except for complicated tasks. For instance, she became confused when she tried to use restrooms outside her home. Her score on the Mini Mental State Examination (MMSE) at the first presentation was 17 with a score of 0 (out of 3) on the recall subscale, which indicated remarkable deficits in recent recall. No remarkable neurological signs were observed. Brain Magnetic Resonance Imaging (MRI) showed no remarkable vascular or mass signs (Figure 1). Single-photon emission computed topography with N-isopropyl[^123^I]-p-iodoamphetamine (IMP-SPECT) was performed at the first presentation (Figure 2). Three-dimensional stereotactic surface projections (3D-SSP) revealed a marked decrease of blood flow in the precuneus and posterior cingulate cortex, which could not be explained by SCZ. However, this is a typical pattern observed in AD.

**Figure 3:**
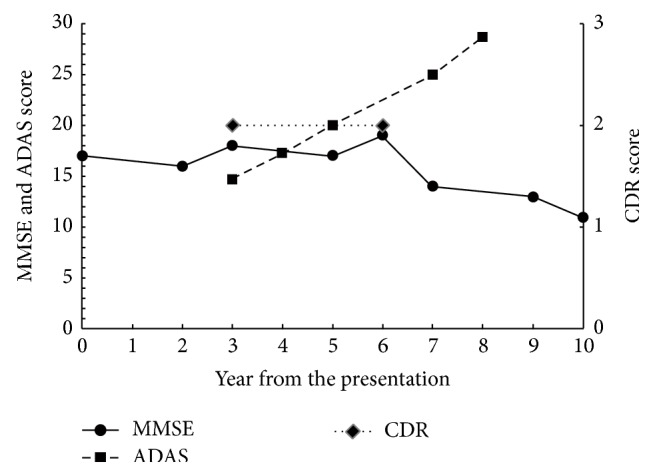
Changes of Mini Mental Scale Examination (MMSE), Alzheimer's Disease Assessment Scale-cognitive subscale (ADAS), and Clinical Dementia Rating (CDR) of patient in Case A. These scores remained stable for 6 years and gradually declined during the last 4 years.

The patient was followed up for ten years. Administration of acetylcholinesterase inhibitors such as donepezil was not considered because it could exacerbate the patient's psychiatric symptoms. Instead, 200 mg of trazodone per day was prescribed. Her cognitive function had not been good but had been stable for six years and gradually declined for the last four years (Figure 3). Since the patient's social activity was very limited even before the onset of AD, she did not interact with anyone except her family members. However, she could accomplish simple tasks such as washing dishes. The MRI images of her brain at the first presentation and six years later are shown in Figure 1. Atrophy of the medial temporal area and dilatation of the lateral ventricles remarkably advanced in six years.

### 2.2. Case B

The patient is a housewife and has a daughter. She had a history of insulin-dependent diabetes mellitus, hypertension, and mild congenital amblyopia. She did not have a history of psychiatric illness until she was in her forties, when she started complaining that someone was slandering her around the neighborhood. Then she was diagnosed with SCZ. The patient's delusions and hallucinations exacerbated intermittently; at all such times, her family took her to a psychiatric clinic and antipsychotics were prescribed. Her psychosis responded well to the medication. However, she often stopped taking medication, following which her symptoms exacerbated. Dose, duration, and the type of medication could not be precisely confirmed because of the lack of medical records. However, her family reported that she was medicated primarily with perphenazine. When the patient was 62 years old, she jumped from the balcony of her apartment and had her femur fractured. Since then, she had been medicated with antipsychotics until she presented to our dementia clinic. At presentation, she was prescribed 8 mg perphenazine, 2 mg biperiden, and 2 mg cloxazolam. This medication controlled her psychotic symptoms for four years.

When the patient was about 66 years old, she was unable to understand doctors' instructions, began to experience memory loss, and often burned the pot while cooking. However, she did not exhibit exacerbation of hallucinations or delusions. She was also unable to manage self-injection of insulin. At 67 years of age, her psychiatrist suspected comorbid dementia and consulted our dementia clinic.

**Figure 4:**
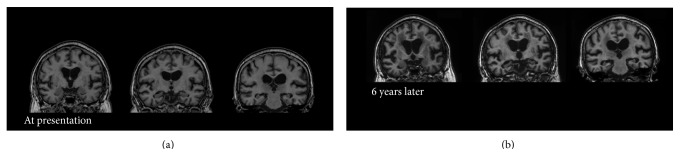
Coronal T1 weighted brain Magnetic Resonance Image (MRI) of the patient in Case B at presentation (a) and 6 years later (b). No mass signs were observed. Low signal intensity in the periventricular white matter suggesting leukoaraiosis was observed at presentation and progressed in 6 years. Diffuse cerebral and hippocampal atrophy, as well as enlargement of the lateral ventricles, which advanced in 6 years, was consistent with comorbid Alzheimer's disease.

**Figure 5:**
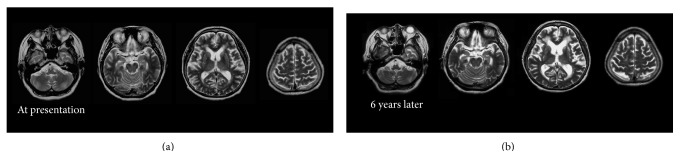
Horizontal T2 weighted brain Magnetic Resonance Image (MRI) of the patient in Case B at presentation (a) and 6 years later (b). Leukoaraiosis at presentation and its progression across 6 years were clearly observed as high signal intensity in the periventricular white matter.

**Figure 6:**
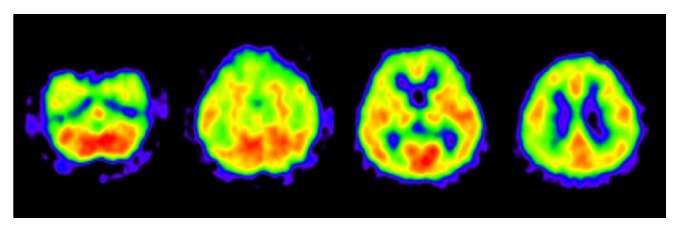
[^15^O]H_2_O positron emission tomography (PET) of Case B. It showed decreased perfusion in the bilateral parietal area and inferior temporal area. Mild decreased perfusion is also observed in the posterior cingulate cortex. These findings suggested comorbid Alzheimer's disease.

**Figure 7:**
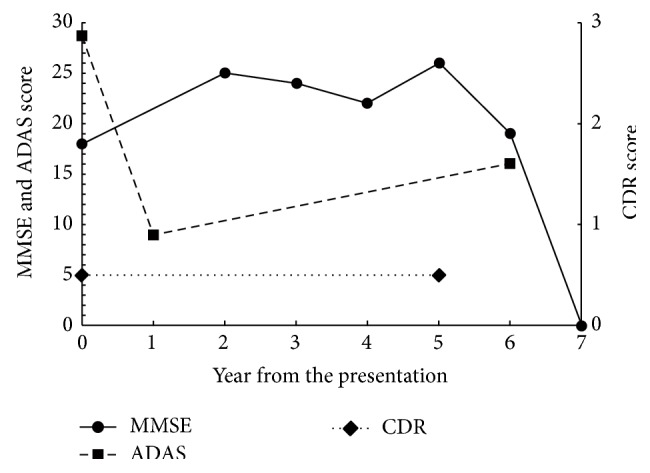
Changes in scores on the Mini Mental Scale Examination (MMSE), Alzheimer's Disease Assessment Scale-cognitive subscale (ADAS), and Clinical Dementia Rating (CDR) of the patient in Case B. The scores remained stable for 6 years and plunged into very severe dementia scores (MMSE score of zero) in the seventh year.

At presentation, the patient could independently perform activities of daily living but suffered severe recent memory deficits. MRI of her brain showed diffuse, medial temporal atrophy and medium periventricular high intensity on T2 intensified images (Figures 4 and 5). [^15^O]H_2_O positron emission tomography (PET) was performed on her in the first year of our follow-up, which showed decreased perfusion in bilateral parietal area and inferior temporal area. Mild decreased perfusion was observed in the posterior cingulate cortex. These findings were consistent with the diagnosis of AD (Figure 6). Considering the risk of relapse of psychotic symptoms, acetylcholinesterase inhibitors were not prescribed. Instead, the aforementioned medications, prescribed before the onset of dementia, were continued. She was followed up for seven years. In the first six years, her cognitive function was stable. The changes in her scores on the MMSE, Alzheimer's Disease Assessment Scale-cognitive subscale (ADAS), and Clinical Dementia Rating (CDR) across time are shown in Figure 7. Her MMSE score at the first presentation was 18, with a score of 0 on the recall subscale. Her initial ADAS score was 25.34 with subscale scores of 7.67 out of 10 and 5 out of 12 for recall and recognition, respectively, which also indicated severe recent memory loss. The MRI images of her brain at the first presentation and six years later are shown in Figures 4 and 5. Neuropsychological indices showed neither increasing nor decreasing trends for six years. In contrast, dilation of lateral ventricle, atrophy of hippocampus, and high intensity of white matter advanced in six years. In the seventh year, her level of cognition and ability to perform activities of daily living plunged without psychosis, apoplexy, or any extreme change in blood sugar levels. She was somnolent and could not follow simple instructions. She was admitted to a convalescent hospital and the follow-up was discontinued.

### 2.3. Case C

**Figure 8:**
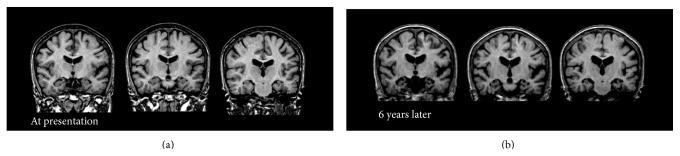
Coronal T1 weighted brain Magnetic Resonance Image (MRI) of the patient in Case C at presentation (a) and 6 years later (b). No remarkable vascular changes or mass signs were observed. Diffuse cerebral and hippocampal atrophy, as well as enlargement of the lateral ventricles, which advanced in 6 years, was consistent with comorbid Alzheimer's disease.

**Figure 9:**
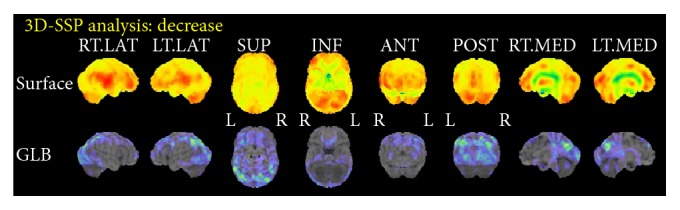
Three-dimensional stereotactic surface projections (3D-SSP) of single-photon emission computed tomography with N-isopropyl[^123^I]-p-iodoamphetamine (IMP-SPECT) of the patient in Case C. Decreased perfusion in the bilateral parietal cortex, precuneus, and posterior cingulate cortex indicated comorbid Alzheimer's disease.

**Figure 10:**
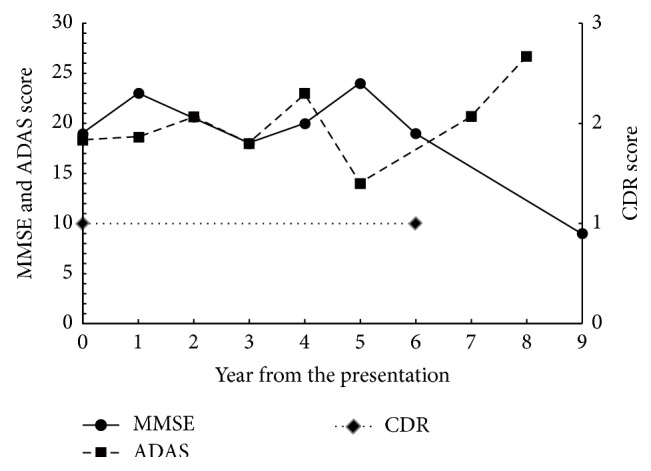
Changes in scores on the Mini Mental Scale Examination (MMSE), Alzheimer's Disease Assessment Scale-cognitive subscale (ADAS), and Clinical Dementia Rating (CDR) of the patient in Case C. These scores remained stable for 6 years and gradually declined for the last 3 years.

The patient was a housewife and had a part-time job. At the age of 40 years, she started complaining about voices from the ceiling of her house. She sometimes talked to strangers on the street but withdrew to home most of the time. Her family brought her to the psychiatric outpatient unit of a general hospital and she was diagnosed with SCZ. Since then, she had been medicated with antipsychotics. The patient's auditory hallucinations lingered despite the treatment; however, she was capable of performing her job as a cleaning worker. At the age of 58, she started saying the same things many times and forgetting appointments. In addition, her psychosis worsened. She was brought to our dementia clinic at the age of 60. Her medication history could not be ascertained because of the lack of medical records. At presentation to our clinic, she was prescribed with 4 mg perospirone and 2 mg flutoprazepam. Although a switch to 8 mg of perphenazine from perospirone and discontinuation of flutoprazepam successfully managed her psychotic symptoms, her memory loss persisted. Scrutiny of her neuropsychological profile showed predominant recent memory loss. Her initial MMSE score was 19 with a score of 0 on the recall subscale. Her initial ADAS score was 18.83 with a score of 7.67 for both recognition and recall subscales. The MRI of her brain showed diffuse cerebral and medial temporal atrophy without remarkable vascular findings (Figure 8). Brain IMP-SPECT showed decreased perfusion in the bilateral parietal cortex, precuneus, and posterior cingulate cortex, which indicated comorbid AD (Figure 9). Donepezil (5 mg) was prescribed in addition to already prescribed 8 mg of perphenazine. The patient was followed up for nine years. Her ability to perform activities of daily living dropped compared with before AD onset but did not change for seven years afterwards. For the first six years, her scores on the MMSE, ADAS, and CDR had not changed significantly and remained between mild and moderate dementia. In the seventh year, her ADAS score did not change either. However, in the eighth year, her ADAS scores increased. In the ninth year, her MMSE score plunged to nine and her CDR to three (Figure 10). Memantine was then added to the prescription. The MRI images of her brain at the first presentation and six years later are shown in Figure 8. Brain atrophy advanced for six years in spite of the relative preservation of cognitive function.

## 3. Discussion

The three cases described above had many characteristics, which could be explained by AD but not SCZ.

All of them had remarkable recent memory loss. Although memory impairment is the most common neuropsychological deficit in SCZ [7], it is controversial whether the memory impairment is a primary amnesic syndrome or a secondary consequence of its global cognitive dysfunction. Although patients with SCZ suffering from memory impairment show defective performance in both recall and recognition tasks, they perform better in recognition, which indicates that their memory impairment could be attributed to deficits in retrieval or registry of memory [8–10]. On the other hand, memory impairment in patients with AD compared with normal subjects was more pronounced in recognition tasks [11]. This neuropsychological profile of AD was applicable to our patients.

MRI images of our patients also provide support for diagnosis of AD. Although substantial reduction of brain volume has been consistently reported in patients with SCZ, the reduction is usually small and not easily observed by visual inspection of images [12]. Progressive reduction of brain volume was also reported in SCZ [13]. However, brain volume decreased mainly in the frontal and temporal lobe and reduced hippocampal volume in SCZ is still controversial [14]. Some researchers argue that although progressive brain tissue decrease in SCZ occurs up to decades after the first symptoms, the annual brain tissue decrease is less than one percent [14]. Our patients showed robust progressive reduction of hippocampal volume and enlargement of lateral ventricles. These findings are consistent with the diagnosis of AD [15].

Abnormal results of brain functional imaging are generally accepted as a characteristic of SCZ. However, most studies reported hypoperfusion of the frontal lobe [16], whereas hypoperfusion of the parietotemporal association cortex, posterior cingulate, and/or precuneus was reported in PET or SPECT of patients with AD [17, 18]. [^15^O]H_2_O PET or IMP-SPECT of our patients revealed hypoperfusion in these areas, which supported the diagnosis of comorbid AD.

Finally, although our patients did not show remarkable cognitive decline in six years, patients A and C presented with cognitive decline in subsequent follow-ups. AD is a more plausible cause for this decline than SCZ.

Collectively, although we did not examine biomarkers such as brain amyloid-beta protein disposition using amyloid PET scans or amyloid-beta levels in the cerebrospinal fluid, our patients had substantial evidence for comorbid AD.

Our patients had some common characteristics. They were all female and married. They lived in the community not in an institution or a hospital. Their social functions were considerably disturbed but their activities of daily living were generally preserved. They were in their 60s at their first presentation to the dementia clinic.

There are several possible explanations for these findings. First, if these patients with SCZ had been severely impaired in activities of daily living, or if they had been living alone, institutionalized, or hospitalized, the symptoms of AD would not have been noticed. Social functions of females suffering from SCZ tend to be preserved well [1], and they have a higher marriage rate than male patients [19]. Symptomatic changes of patients living with their family are more likely to attract attention than similar changes in institutionalized patients. These factors could explain the demographics characteristics of our patients, that is, female and married. Second, the number of patients with AD in their fifties or younger is relatively small. On the other hand, in patients in their 70s or older, cognitive decline is unlikely to be regarded as pathological, especially in patients with existing cognitive impairment such as that observed in SCZ. This accounts for the fact that our patients with SCZ were in their 60s at the first presentation to our dementia clinic.

Our results indicated slower cognitive decline in patients with AD and comorbid SCZ compared with average patients with AD. The reported rate of decline in the MMSE score in AD ranges between 0.9 and 4.4 [20–25]. The annual decline of MMSE scores in AD differs widely among studies, which is, at least in part, explained by the fact MMSE scores decline slowly in mild (with MMSE scores of 20 or more) or very severe (with MMSE scores of five or less) dementia [23]. Patients with an initial MMSE score of about 18, which is the initial score of our patients, are expected to show a robust decline. There was wide variability in the individual rate of decline in MMSE scores and the scores are not reliable enough for detection of cognitive change in a short period. However, the validity of the measurements improved with observation for three or more years [21, 26]. Our six-year follow-up is long enough to compensate for this large measurement error and substantial variation in the change of MMSE scores. In summation, it could be concluded that our patients showed a significantly slower decline of MMSE scores than general patients with AD.

Because the patient described in Case A had not undergone the test until the fourth year, our data of a longitudinal change in ADAS scores is incomplete. However, the ADAS scores of our patients deteriorated more slowly than that reported in previous studies, which reported an annual rate change of 0.9 to 9.7 [25, 27–30]. In addition to changes in the MMSE score, the annual rate change of ADAS is more rapid in patients with moderate dementia than in those with mild or severe forms [27, 30]. The ADAS score of the patient in Case A increased from 14.7 (three years after the presentation) to 25.0 (seven years later) yielding 2.6-point mean annual rate of change. The score of the patient in Case B decreased from 28.7 (year of presentation) to 16 (six years later) and that of the patient in Case C only modestly increased from 18.3 (year of the presentation) to 20.7 (seven years later) yielding 2.1- and 0.3-point mean annual rate of change, respectively. These changes of ADAS scores in our patients were obviously slower than the previously reported changes in patients with AD. These data strengthen the notion that our patients showed significantly slower cognitive deterioration despite substantial evidence of comorbid AD.

As mentioned before, studies on comorbidity of SCZ and AD are rare despite the high prevalence of both. As far as we know, this is the first follow-up study of multiple patients with AD and premorbid SCZ with neuropsychological and neuroimaging assessments. Prohovnik et al. reported higher Alzheimer-type neuropathology in the elderly with SCZ [5]. However, other researchers argued against such a conclusion [6]. It is not clear whether SCZ is a risk factor for or a protective factor against AD.

Our results suggest that comorbid SCZ slowed cognitive decline after the onset of AD. The most plausible explanation is poor cognitive reserve of patients with SCZ. It was reported that people with a greater cognitive reserve such as those with higher education were resilient against AD neuropathology [31, 32]. However, once they started to present with the dementia syndrome, higher education associated with more rapid global decline [33]. In other words, people with poor cognitive reserve present dementia syndrome earlier with less AD neuropathology and show slower progression after the onset than those with greater cognitive reserve. Many investigators have described poor cognitive performance in SCZ [3, 4], which is in agreement with the cognitive reserve hypothesis in the comorbidity of SCZ and AD. Dwork et al. investigated neuritic senile plaques and neurofibrillary tangles of elderly institutionalized patients including 66 SCZ and 36 dementia patients. They found that only 8% of patients with SCZ satisfied neuropathological criteria of AD even though 68% of patients with SCZ had definite cognitive impairment [34]. However, among their SCZ subjects without AD, cognitive impairment was associated with higher levels of plaques and tangles. They concluded that an association of mild Alzheimer-type pathology with definite cognitive impairment was unique to SCZ and that it is a manifestation of poor cognitive reserve in SCZ. Their finding also supports the cognitive reserve hypothesis and thus explains our results.

Although poor cognitive reserve and earlier presentation of dementia could explain our results, the difference of progression of cognitive decline between our patients and patients with AD in general is much greater than that due to education. Therefore, some neurostructural factors in SCZ brain could be protective against the progression of AD neuropathology; this deserves further investigation.

Currently, aging of the human population is an issue not only in economically developed countries but also in developing countries. The number of patients with schizophrenia in their old age is increasing. More than 15 years ago, it became apparent that we need to improve our understanding of the neurobiological and psychosocial factors underlying late life schizophrenia, as well as to develop more effective and safer pharmacological, psychosocial, and cognitive behavioral treatments [35]. Nevertheless, the studies focused on pathology, clinical course, and treatment of late life schizophrenia are still far from sufficient. There are a few anecdotal case reports on geriatric comorbidities of late life schizophrenia and their management and systematic investigations with clear objectives and statistical analysis are rare. To the best of our knowledge, there have not been any reports that followed up schizophrenia patients with comorbid Alzheimer's disease.

Many questions remain to be answered regarding the comorbidity of SCZ and AD. For instance, effectiveness of acetylcholinesterase inhibitors or memantine for AD with comorbid SCZ is unclear, especially considering that many patients with SCZ receive anticholinergics in addition to antipsychotics. It is not clear whether administration of acetylcholinesterase inhibitor has a substantial risk for exacerbation of SCZ symptoms. In our patients, the prescription of these medications was decided based on clinical judgments of the doctors at the patients' first presentation, which might be arbitrary.

The effect of antipsychotics on the onset of AD in SCZ and cognitive decline after the onset of AD is also unclear. Antipsychotic medications slightly improve cognitive function in SCZ [36] whereas they are associated with cognitive decline in dementia [37]. Among our patients, the one described in Case A had discontinued treatment shortly after the onset of SCZ and was not prescribed any antipsychotics at the time of presentation to our clinic and remained free of antipsychotics after the onset of AD. The patient in Case B had been treated with antipsychotics after the onset of SCZ with frequent interruptions. Medication was continued after the onset of AD and the compliance was improved because her family started managing the medication for her. The patient in Case C seems to have been compliant with respect to medication since the onset of SCZ and the medication was continued after the onset of AD. It is difficult to find any relationships between these different levels of compliance with antipsychotics before and after the onset of AD and the onset or the course of cognitive decline in our patients. Our sample size is too small to draw any conclusions. Further research with an adequate sample size is required.

It is also not clear whether the onset of AD in SCZ could precipitate psychotic symptoms. Psychotic symptoms of the patient in Case C relapsed simultaneously with the signs of AD whereas the others showed AD symptoms without exacerbation of psychosis.

Our study has several limitations. Our patients were not recruited systematically and the number of patients was too small. We did not quantify the morphological or functional changes observed using brain images. In addition, we did not measure biomarkers such as brain amyloid-beta deposition.

Nevertheless, our data suggest the need for further research to elucidate the interaction between SCZ and AD. The patients with AD and comorbid SCZ should be recruited in larger numbers systematically by screening elder patients with SCZ. Neuropsychological and neuroimaging data should be collected broadly, regularly, and quantitatively. Biomarkers such as levels of brain amyloid-beta protein deposition should be tested using amyloid PET scan or by direct measurement of amyloid beta in the cerebrospinal fluid.

In conclusion, we studied three patients with SCZ who developed AD in their 60s. Their cognitive decline was slower than that of the average patients with AD, even though many neuropsychological and neuroimaging characteristics were explained by AD but not SCZ. The cognitive reserve model could partially account for this discrepancy; however, the possibility of interactions between SCZ and AD neuropathology remains open. Our findings warrant further investigation into the clinical and biological interactions between SCZ and AD.

This study was performed at the Department of Aging Brain and Cognition, Hyogo Brain and Heart Center at Himeji, 520 Saisho-ko, Himeji, Hyogo, Japan.
